# Recurrence after Thyroid Operations

**Published:** 1917-03

**Authors:** S. P. Beebe

**Affiliations:** New York


### Recurrence After Thyroid Operations.

BY S. P. BEEBE, PH. D., M. D., NEW YORK.

The treatment of thyroid disease has been the subject of an increasing amount of discussion during recent years. Ten years ago, when the writer first became interested in the matter, very few men, either as investigators or as clinicians, gave time or thought in this direction, and the articles devoted to this phase of medicine were few in number. At present scarcely any medical journal is without some original contribution or comprehensive review. New understanding of the nature of the disease process in this gland has led to improved methods of treatment and diagnosis. Ten years ago few operations for hyperthyroidism were performed in this country. At present, however, in at least one great surgical clinic, operation on the thyroid gland is performed on more patients than are operated on for any other single disease. This experience has been to some degree duplicated in other places so that it would seem to be a surgical procedure well established in the confidence of the medical profession. Not infrequently statistical reports are made to show the beneficial results obtained and the low mortality that is possible in such work when properly done. Recently the Swiss Surgical Society has asked for complete reports to be sent giving all the data with respect to such operations in order to form a collective experience table based on the results obtained in all cases of radical operation on goiter. It is indeed desirable to have such a collective picture of thyroid surgery. In the earlier work the surgeon was evidently concerned more with the mortality incident to the operation than to any other one feature. And such a concern was in every way proper. There is a kind of finality about an operative death that admits of little argument. The technique of the operation as well as the surgical judgment with respect to goiter were at first so little developed as to make operative mortality a most important matter for consideration. The experience of the last ten years has led to great improvement in these respects so that in certain surgical clinics operation on the thyroid gland is now accomplished with a very small immediate risk to the patient, but this very fact has led to unfortunate results. Unfortunate because, relying upon the favorable mortality rates shown in the best clinics, many surgeons who have neither the technique nor the surgical common sense necessary for this work urge and perform operation often with disastrous results. The skill and judgment necessary to do successful thyroid surgery have, as a rule, been obtained at the cost of many unfortunate victims. The writer is firmly convinced that it is just as necessary to have a thorough knowledge of the disease to do successful thyroid surgery as is required to treat the condition medically. Thyroid surgery requires a surgical specialist just as much as surgery of the mastoid, the Gasserian ganglion, or the rectum. With the improvement in the mortality rate, the therapeutic benefits of the operation which were at first assumed have been more closely studied. Statistical tables prepared from answers to a series of questions forwarded by mail to the operated patient form the basis for statements concerning the percentages cured, markedly relieved, etc., as a result of the surgical procedure. Such tables must necessarily be misleading and inaccurate because the patient's own judgment regarding his condition is not always reliable. Hyperthyroidism often proceeds to such a degree of severity as to seriously impair the efficiency of its victim without his being aware of it, and while the patient may accurately report the state of his mind with respect to himself, he is not the most suitable person to decide with accuracy the state of his thyroid activity. The writer has seen no statistics based upon a careful physical examination of the operated patient some months or years after the operation by a physician thoroughly acquainted with the disease. He is confident that the results of such an examination would not afford as favorable a showing for thyroid operations as are now published on the basis of correspondence. One of the most prominent and successful of the operators on the thyroid gland stated in a public discussion of this subject some years ago that in his experience he had never seen a complete operative cure in a case of exophthalmic goiter. This statement comes from one of the most enthusiastic advocates of the operative treatment. It is in agreement with the writer's experience. The number of patients subjected to various thyroid operations in this country during the last ten years must form a total of many thousand; I believe 25,000 a conservative estimate. Surgical skill and experience have eliminated so much of the earlier danger that this number is likely to be far exceeded during the next decade. In spite of the brilliant surgical results often obtained, the surgical treatment has not won the entire confidence of the profession. There is a sharp difference of opinion between many physicians and surgeons regarding the efficacy of various therapeutic procedures. The physician has been held responsible for what is termed the "medical death" in that he did not submit his patient to the surgeon at an early period of the disease, but in justice it should be stated that many patients do not apply for treatment until the condition is well advanced. On the other hand, the surgeon is criticized for much unnecessary surgery and for not using proper care in respect to preparation of the patient for the operation, and in the after care of the patient. The feeling between the two branches of the profession regarding this matter is acute; and in sharp contrast to the harmony of mind respecting other surgical conditions; appendicitis for example. Obviously such a state of affairs has its origin in differing viewpoints, and in differing experience.

In considering this difference of opinion, the writer feels that there is one phase of the matter which has not received sufficient emphasis from either party to the discussion. This is the question of recurrence following operation. As a rule, the surgeon says very little about recurrence in his statistics; it is, nevertheless, a factor of much significance in making a decision regarding the character of the surgical procedure and the after care of the patient. The writer is not able to give any accurate figures regarding the frequency of recurrence after partial thyroidectomy, but is able to state that many patients have come under his care for this condition during the last few years.

The figures that are given by different operators with respect to recurrence vary from 20 to 40 per cent of the operated cases. This variation is no doubt in some degree due to the different types of cases selected for operation by different operators and to the kind of procedure which they follow in dealing with the patient. Recurrence should properly be limited to increased growth of gland tissue and redevelopment of symptoms following partial thyroidectomy and not to changes which are a sequel to ligation operations. The statistics now to be gathered by the Swiss Surgical Society probably give more accurate figures than have heretofore been available regarding the frequency of recurrence, but from the statistics and estimates that are now available it appears that approximately 30 per cent of the patients subjected to partial thyroidectomy show well marked recurrence of both gland and symptoms.

For the further purposes of this discussion, reference will be made only to recurrence following operations for severe hyperthyroidism, including those patients having exophthalmic goiter, although the writer has seen many cases of recurrence following thyroidectomy in cases of so-called simple goiter without toxic symptoms. As a further basis for the discussion, the writer states that he has had 21 patients operated on (partial thyroidectomy) during the last five years for hyperthyroidism, but in not a single case was it possible to dismiss the patient in a satisfactory condition of health until they had been subsequently treated medically for a period of from four months to a year and a half. In eleven of these operated cases there was marked increase in size of the remaining thyroid tissue, accompanied by exacerbations of the disease. This experience coupled with the numerous cases that have been seen for the first time with a well developed recurrence of both gland and symptoms has convinced the writer that from the standpoint of both the medical adviser and the patient a fair examination and presentation of the available facts should be permitted.

The operative procedures most commonly practiced in this country are two: ligation of one or more arteries supplying blood to the gland, and partial thyroidectomy. The ligation operations vary somewhat with different operators. Some men ligate the arteries after separating them from the accompanying veins; others prefer to ligate both arteries and veins, while the pole ligation includes all the structures entering or leaving the upper or lower pole of the gland. This operation had its origin in the belief that the partial interruption of the blood supply would permit less active functioning of the hyperactive gland tissue. Such an assumption has some basis in fact, but in no case is there attempted a complete occlusion of the blood supply to the whole gland. We know that the blood supply to the thyroid is not arranged as a terminal system such as is found in the kidney. Consequently this operation reduces temporarily the total blood supply to the whole gland but does not at any time completely put out of commission any portion of the gland tissue such as would be the case in the kidney if the branch of the renal artery were ligated. The collateral circulation can and does frequently develop to such a degree as to restore conditions on nearly the same basis as before. In many instances a ligation operation properly done starves the gland enough to reduce temporarily the degree of hyperthyroidism, and permits an otherwise impossible partial thyroidectomy. Simultaneous ligation of all four thyroid arteries has been attempted in a few instances but with disastrous results. In one case known to the writer this procedure was followed by the development of acute tetany and death. In the writer's opinion, there is reason to believe that the benefits of the ligation operation are to a large degree due to the simultaneous cutting of a portion of the nerve supply to the gland. The experimental work of the last few years has demonstrated beyond reasonable doubt a nerve control of thyroid function. The nerves enter the gland in close association with the arteries. Ligation of the artery includes the nerve with the result of blocking further impulses to at least a portion of the gland. The collateral circulation may very promptly re-establish former conditions of blood supply, but the regeneration of the nerve is admittedly a much slower matter. There is no experimental evidence to demonstrate in this particular instance whether it may or may not occur. As an instance of the beneficial effects of the ligation operation with a later relapse, the writer cites the following case history:

1. Patient was a woman 29 years of age. Eighteen months before presenting herself for serum treatment she had a ligation of both superior thyroid arteries by a competent surgeon. Previous to the operation she had all the symptoms of a fairly severe hyperthyroidism, the exophthalmos of the right eye being more pronounced than the left. She remained in the hospital for ten days at the time of the operation, she gained in weight and strength, was much less nervous, not troubled by her heart, and for a period of nearly one year she had the activity and responsibility of a normal woman, and was assured by her medical adviser of her complete recovery. During the last nine months there has been a slow and steady growth of her gland, she has lost nearly thirty pounds in weight during this time, she has a rapid, irregular pulse, 130-145, is very nervous, has marked muscular tremor, and the exophthalmos of the right eye is much more pronounced than the left. In spite of her improvement and good condition during the first ten months following the operation, she is now in a worse condition than at any time previously.

In this case the improvement following the operation was pronounced; in fact, it reached such a degree that for a few months both she and the surgeon considered a complete recovery to have been established. The period of improvement lasted for a longer time than might reasonably be required to establish an efficient collateral circulation through the gland, and the question arises whether the development of symptoms and increased growth may not have been coincident with the regeneration of nerve supply. The attitude taken toward different patients with respect to the purpose of a ligation operation is subject to variation. In some instances it is regarded as an end in itself, and after the operation a more or less complete period of rest with various accompanying medical measures is considered a sufficient and safe method of procedure to restore the patient to health. In other cases it is used principally as a preparation for the more severe shock of a partial thyroidectomy, and in these cases a second operation follows the first, oftentimes at an interval of ten days to two weeks. Occasionally a period of two or three months is allowed to elapse before the final extirpation of the gland. From the writer's observation of a considerable number of cases that have been subjected to a ligation operation, he feels convinced that in itself, without further operative procedure, or without suitable and adequate continued medical treatment, it does not give permanent relief to the patient. The case cited is a further example of its effects.

The second type of operation, partial thyroidectomy, is usually relied upon by the surgeon to afford satisfactory, lasting relief to the patient. It seems to be a reasonable proposition to reduce the hyperactivity of the gland by removing a large portion of the gland itself. However, this is an instance in which surgical judgment needs careful cultivation to decide how much gland may be safely removed to leave the patient a sufficient amount of secreting tissue to answer the physiological needs of the body. If too much is removed, there is a very grave possibility of troublesome hyperthyroid conditions developing later. If not enough is removed, the symptoms may be relieved very markedly, the patient, at least for a time, being restored to a fair condition of health, but the remaining portion of the lobe may very readily change the intensity of its activity to the detriment of the future health of the patient. The right lobe of the thyroid is in most instances the first to increase in size, and it usually develops to a much larger size than the left lobe. Consequently, partial thyroidectomy in the majority of instances means the removal of the right lobe and isthmus with the possible ligation of the inferior thyroid arteries on the left side. It is not to be denied that very satisfactory effects are very frequently obtained by such on operation. It is equally certain that the remaining left lobe not infrequently increases in size and activity to such a degree that from one to five years after the original operation the patient is in nearly, if not quite, as desperate a condition of health as before any operative procedure was begun. The mere removal of a portion of the thyroid tissue does not serve in itself to remedy the fundamental physiological faults which have permitted or stimulated the gland to its increased size and function in the beginning. The precise reasons why the thyroid takes on this unusual development is not thoroughly understood. In a considerable percentage of cases it seems to be a reaction to chronic infection; in others, it may be a reaction to excessive work or emotional strain.

Additional study has demonstrated that no insignificant percentage of the so-called simple goiters eventually are subject to a change in the activities which they present and develop toxic manifestations and in all respect demand remedial action quite as imperatively as those that have been definitely toxic or overactive in their inception. Physicians have regarded too lightly the so-called simple enlargements of the thyroid which have not shown active and urgent symptoms from the beginning. In tracing back the history of patients with well marked classic hyperthyroidism, accompanied by exophthalmos, the writer frequently finds that the first enlargement of the gland was noted at a comparatively early age, from the twelfth to the sixteenth year, and at that period no significance was attached to this enlargement.

In fact, the patient has been frequently advised by the medical attendant to pay no attention to the gland. The increase in size has been a very gradual one, the patient has remained in good health seemingly until the age of 20 to 25, when characteristic symptoms have developed to such a degree as to make corrective measures imperative. These patients are in somewhat sharp contrast to those in whom the gland enlargement is sudden and rapid, with an acute development of the symptoms of the disease. For many years the condition has been to some extent latent and because no really serious impairment of health was evident no attention has been paid to the gland. These changes in the activity of the gland, as well as the increase in size of the gland, are in the nature of a reaction to influences which have originated in other portions of the body and have reached the gland through the medium of the blood stream or the nervous system.

The writer has made many experiments to test physiologic activity of extracts prepared from the so-called simple goiters, and has found that these extracts are in every respect similar to those obtained from normal glands in proportion to their iodin content. In making this statement, we are not considering cystic glands or those that have developed various forms of degeneration. As a rule, the iodin content of the enlarged simple gland is per gram of gland less than that found in a gland of normal size and histology. Such a gland has in fact accumulated a storehouse of material which is physiologically active in proportion to its iodin content. Such a gland causes no toxic symptoms, not because it does not contain within itself a secretion capable of provoking the symptoms of hyperthyroidism, but because the material has been retained within the gland itself and it has needed only the proper stimulus to cause its discharge into the general circulation to set up the train of symptoms which are recognized as requiring immediate correction.

It may be argued that in removing a gland which is the source of increased secretion only diseased tissue is being removed, and no particular concern need be paid to the future welfare of the patient. It is, nevertheless, true that a gland which at one period in its history is markedly overactive may if left to itself pass through a series of changes which result in a partial destruction of the gland and a very marked impairment in its functional activity so that later a defective and insufficient thyroid function arises. The surgical removal of the major portion of the gland certainly puts out of commission that amount of active gland tissue but may leave the patient in future years with a less active thyroid function than the condition of health demands, so that we are now seeing some of the later results of thyroid surgery in such conditions.

It is possible by medical means to relieve a very large percentage of the patients having overactive glands; not only to relieve them temporarily but to leave them in a condition of health and with the enlarged thyroid reduced in size to normal limits and in function able to do the work required.

The writer has had few opportunities to study histologically the thyroid gland, which has at one period in its history been enlarged to eight or ten times its normal size and the important link in a change of events causing a severe exophthalmic goiter. A patient who has recovered without operation from such an experience naturally prefers to retain the gland, and in fact it has been possible to obtain only one such gland for histologic study. This gland showed on section an increased amount of connective tissue, but had an abundant supply of alveoli lined with normal secreting epithelium and filled with a normal staining colloid.

If the surgeon relies alone upon the partial thyroidectomy to relieve the patient permanently and gives no study to determine the stimulating causes of the hyperfunction and does not seek to remedy these faults, his duty to the patient is only partially met and ultimate disappointment will result in many instances.

As stated previously in this article, the writer is not able to quote a large mass of statistics with respect to the frequency of recurrence of the tumor and symptoms following partial thyroidectomy for the reason that he has not had many patients subjected to operation, and naturally comes in contact only with the recurrent cases that have been operated on by other men. The fact, however, that he has such cases constantly under treatment, and that a very considerable group of them have been under treatment during the last few years, indicates a condition of affairs which he believes requires emphasis. Recurrence may take place rapidly. The surgeon prefers, as does the physician, to reach his patient in the early development of the disease. In fact, some surgeons advocate the immediate removal of a large portion of any gland found enlarged regardless of any untoward effects which may be evident upon the patient's health at that particular time. This attitude is maintained because of the probable difficulties that will ensue in the future if such a course is not taken. The surgeon has criticized the physician severely for not subjecting to immediate operation those patients, mostly young women between 18 and 28 years of age, who are discovered to have the beginnings of exophthalmic goiter. As an instance of what may follow if this advice is universally followed, the case cited herewith is an example.

A young woman, 22 years of age, college student, was brought to a physician with a well developed thyroid enlargement, mostly in the right lobe and isthmus of the gland. The enlargement had taken place rapidly during the last three months. There had been a loss of weight of approximately 12 pounds during this period. The patient was nervous, sleepless, had headaches, characteristic overacting heart, with a pulse rate of 120, marked physical weakness, complete interruption of the menstrual periods during this period and a mild exophthalmos. The diagnosis was not difficult. The patient's family decided on an immediate operation, which was performed by a competent surgeon and consisted of the removal of the right lobe and isthmus of the gland under local anesthesia. The patient improved markedly following this operation; she was in the hospital altogether a period of a little over two weeks, following which time she went to a quiet place in the country for a prolonged rest. The remaining left lobe was apparently quiescent for a period of several months; she gained in weight and the disturbing toxic symptoms were apparently well under control. After a period of ten months, following the operation, she returned to the writer's observation with the left lobe fully as large as the right lobe had formerly been, with all the characteristic manifestations of the disease as well marked as they had been prior to the operation. The surgeon suggested the removal of an additional portion of the left lobe. This was refused by the family, and medical treatment was instituted and continued for a period of a year. The final result was that the large lobe was so much reduced in size as to be no longer observed as a tumor in the neck, although its outlines could be determined by palpation some months after this time. The disagreeable and annoying hyperthyroid symptoms were completely relieved, and during the last three and a half years this patient has been in excellent health, with no further treatment of any kind. This is an instance of a typical recurrence taking place when operation was performed early in the development of the disease. Operation alone, with a period of rest in the country, had been relied upon to correct the condition. The relief immediately following the operation was complete, but the marked typical recurrence later on indicated that those factors that had been operative in producing the development of the gland with its consequent toxic manifestations had in no way been controlled by the simple removal of a considerable portion of the affected structure.

The writer is convinced that the frequency of recurrence, together with the incomplete relief afforded in a large percentage of cases, demands that the patient have the benefit of something more than the simple ligation of the blood supply or a partial extirpation of the gland, in order to produce a final and permanent condition of good health. The surgeon, as a rule, does not have the experience, time or the attitude toward the problem of medical treatment that is characteristic of the physician. Evidence is not wanting that with increasing experience many surgeons appreciate in a way they did not in the beginning of their thyroid work the necessity for medical supervision and direction of the patient following operation, but the writer is convinced that the best interests of the patient in dealing with so intricate and complex a problem will best be served by the co-operation of a physician and surgeon who have a sufficient amount of respect for each other's integrity and ability to enable them to work in harmony in deciding the course to be followed in any given case.

